# Clinical utility of target-based next-generation sequencing for drug-resistant TB

**DOI:** 10.5588/ijtld.22.0138

**Published:** 2023-01-01

**Authors:** H. Mansoor, N. Hirani, V. Chavan, M. Das, J. Sharma, M. Bharati, V. Oswal, A. Iyer, M. Morales, A. Joshi, G. Ferlazzo, P. Isaakidis, Z. Ndlovu, K. England

**Affiliations:** 1 Médecins Sans Frontières, Mumbai, India; 2 Grant Medical College, Sir Jamshedjee Jeejebhoy Group of Hospitals, Mumbai, India; 3 National TB Elimination Programme, Mumbai, India; 4 Southern Africa Medical Unit, Médecins Sans Frontières, Cape Town, South Africa; 5 Clinical and Molecular Epidemiology Unit, Department of Hygiene and Epidemiology, University of Ioannina School of Medicine, Ioannina, Greece; 6 Division of Epidemiology and Biostatistics, Department of Global Health, Faculty of Medicine and Health Sciences, Stellenbosch University, Cape Town, South Africa; 7 Independent Consultant, Infectious Disease Microbiologist, Honolulu, Hawaii, USA

**Keywords:** tuberculosis, diagnosis, genome sequencing

## Abstract

**BACKGROUND:**

In high TB burden countries, access to drug susceptibility testing is a major bottleneck. Targeted next-generation sequencing (tNGS) is a promising technology for rapid resistance detection. This study assessed the role of tNGS for the diagnosis of drug-resistant TB (DR-TB).

**METHODS:**

A total of 161 samples from bacteriologically confirmed TB cases were subjected to tNGS using the Deeplex^®^ Myc-TB kit and sequenced using the MiSeq platform. These samples were also processed for conventional phenotypic DST (pDST) using 13 drugs on Mycobacteria Growth Indicator Tube and line-probe assays (MTBDR*plus* and MTBDR*sl*).

**RESULTS:**

There were 146 DR-TB and 15 drug-susceptible TB (DS-TB) samples. About 70% of patients with DR-TB had no previous TB treatment history. Overall, 88.2% had rifampicin-resistant/multidrug-resistant TB (RR/MDR-TB), 58.5% pre-extensively drug-resistant TB (pre-XDR-TB) and 9.2% had XDR-TB as defined by the WHO (2020). Around 8% (*n =* 13) of samples were non-culturable; however, identified 8 were resistant to first and second-line drugs using tNGS. Resistance frequency was similar across methods, with discordance in drugs less reliable using pDST or with limited mutational representation within databases. Sensitivities were aligned with literature reports for most drugs. We observed 10% heteroresistance, while 75% of strains were of Lineages 2 and 3.

**CONCLUSIONS:**

Programme data supported tNGS in the diagnosis of DR-TB for early treatment using individualised regimens.

## Abstract (fr)

**CONTEXTE:**

Dans les pays où la TB est très répandue, l’accès aux tests de sensibilité aux médicaments constitue un obstacle majeur. Le séquençage ciblé de nouvelle génération (tNGS) est une technologie prometteuse pour la détection rapide de la résistance. Cette étude a évalué le rôle de cette technique dans le diagnostic de la TB résistante aux médicaments (DR-TB).

**MÉTHODES:**

Au total, 161 échantillons provenant de cas de TB confirmés par des analyses bactériologiques ont été soumis à un tNGS à l’aide du kit Deeplex® Myc-TB et séquencés sur la plateforme MiSeq. Ces échantillons ont également été traités pour une DST phénotypique conventionnelle (pDST) utilisant 13 médicaments sur le tube indicateur de croissance des mycobactéries et des tests à sonde linéaire (MTBDR*plus* et MTBDR*sl*).

**RÉSULTATS:**

Il y avait 146 échantillons de DR-TB et 15 échantillons de TB-susceptible aux médicaments (DS-TB). Environ 70% des patients atteints de DR-TB n’avaient pas d’antécédents de traitement antituberculeux. Dans l’ensemble, 88,2% d’entre eux présentaient une TB résistante à la rifampicine/multirésistante (RR/MDR-TB), 58,5% une TB pré-résistante aux médicaments (pre-XDR-TB) et 9,2% une XDR-TB selon la définition de l’OMS (2020). Environ 8% (*n* = 13) des échantillons n’étaient pas cultivables ; toutefois, 8 ont été identifiés comme étant résistants aux médicaments de première et de deuxième ligne par la méthode tNGS. La fréquence de la résistance était similaire d’une méthode à l’autre, avec une discordance dans les médicaments moins fiables en utilisant la pDST ou avec une représentation mutationnelle limitée dans les bases de données. Les sensibilités étaient alignées avec les rapports de la littérature pour la plupart des médicaments. Nous avons observé 10% d’hétérorésistance, tandis que 75% des souches étaient de lignées 2 et 3.

**CONCLUSIONS:**

Les données du programme ont permis d’appuyer le tNGS dans le diagnostic de la DR-TB pour un traitement précoce à l’aide de régimes individualisés.

Nearly half a million people are affected by rifampicin-resistant TB (RR-TB) worldwide; of these, 78% have multidrug-resistant TB (MDR-TB).^1^ In 2020, 10 countries collectively accounted for 74% of the global gap between estimated TB incidence and the number diagnosed and reported with TB. India is one of the top three contributors (24%)^2^ due to a combination of underreporting and under diagnosis, and accounts for one quarter of the world’s drug-resistant TB (DR-TB) cases, including FQ-resistant cases at 21.8% of MDR-TB patients.^3^ Dalal et al. reported that pre-XDR-TB (56.8%) was more common than MDR-TB (24%) in Mumbai, India.^4^

Conventional diagnosis is based on phenotypic drug susceptibility testing (pDST) which has significant drawbacks (delayed results, limited accuracy, poor reproducibility and inability to determine mixed infections or heteroresistance). Current rapid molecular tests that detect resistance to key first- and second-line drugs have, however, revolutionised TB diagnosis. These assays (e.g., GeneXpert^®^ MTB/RIF; Cepheid, Sunnyvale, CA, USA; and GenoType^®^ MTBDR*plus* and GenoType^®^ MTBDR*sl* line-probe assays [LPAs]; Hain Lifescience, Nehren, Germany) have significantly reduced the time to treatment initiation for TB/DR-TB patients. However, none of these assays can be used to detect the full range of resistance for all currently used anti-TB drugs.

Interest in the use of targeted next-generation sequencing (tNGS) for DR-TB diagnosis is increasing.^5^,^6^ Methods based on direct sputum for analysis make tNGS attractive due to its potential for rapid turnaround of results. The targeted approach focuses on known gene targets where resistance-conferring mutations commonly occur. Thus, resistance predictions are possible for most anti-TB drugs in a single sequence, which facilitates the establishment of a complete “resistotype” for each sample. Furthermore, levels of micro/hetero-resistance and detection of mixed infections provide additional information to better guide case management.^7^

The present study explored the utility of tNGS as a diagnostic tool and the potential for its routine use in DR-TB case management.

## METHODS

### Study design

This was a descriptive study conducted between October 2019 and September 2020.

### Study setting

Mumbai, India, a densely populated city in Maharashtra,^8^ is a hot spot for DR-TB; 24% of newly diagnosed and 41% of previously treated MDR-TB cases are never treated.^9^ Various studies have reported fluoroquinolone (FQ) resistance rates of nearly 60% among MDR-TB cases.^4^

The study was conducted at the Shatabdi Hospital, Mumbai, India, under India’s National TB Elimination Programme (NTEP), which is supported by Médecins Sans Frontières (MSF; Paris, France) for comprehensive DR-TB patient care.^8^

### Laboratory procedures

Two sputum samples were collected from each patient with presumptive TB. Spot specimens from MTB-positive on Xpert MTB/Rif were sent to Jamshedjee Jeejebhoy (JJ) Group of Hospital TB laboratories for resistance profiling. The resistance profiling of TB drugs was carried out using GenoScreen MycTB (GenoScreen, Lille, France) tNGS, MTBDR*plus* or MTBDR*sl* LPA and BD MGIT™ (BD, Franklin Lakes, NJ, USA) pDST for the following 13 anti-TB drugs: isoniazid (INH), rifampicin (RIF), ethambutol (EMB), pyrazinamide (PZA), streptomycin (SM), kanamycin (KM), amikacin (AMK), capreomycin (CPM), levofloxacin (LVX), moxifloxacin (MFX), ethionamide (ETH), linezolid (LZD) and clofazimine (CFZ). The critical concentrations used in the tests were based on NTEP guidelines (Table 1).

**Table 1:** GenoScreen Deeplex® MycTB tNGS targets amplified for tNGS by drug compound and critical concentration used in pDST *

Drug	Gene targets	Critical concentration (μg/mL)
INH	*kat*G, *inh*A, *fab*G1, *ahp*C	0.1
RIF	*rpo*B	1.0
EMB	*emb*B	5.0
PZA	*pnc*A	100.0
SM	*rrs, rps*L*, gid*B	1.0
FQ	*gyr*A*, gyr*B	LVX (1.0), MFX-low (0.25, MFX-high (1.0)
KM	*eis, rrs*	2.5
AMK	*rrs*	1.0
CPM	*rrs, tly*A	2.5
ETH	*eth*A*, fab*G1*, inh*A	5.0
LZD	*rpl*C*, rrl*	1.0
CFZ	*rv0678*	1.0

* Bedaquiline pDST was not routine at the time of the study and was therefore not performed.

tNGS = targeted next-generation sequencing; pDST = phenotypic drug susceptibility testing; INH=isoniazid; RIF = rifampicin; EMB = ethambutol; PZA = pyrazinamide; SM = streptomycin; FQ = fluoroquinolone; KM = kanamycin; AMK = amikacin; CPM = capreomycin; ETH = ethionamide; LZD = linezolid; CFZ = clofazimine.

**Figure 1:**
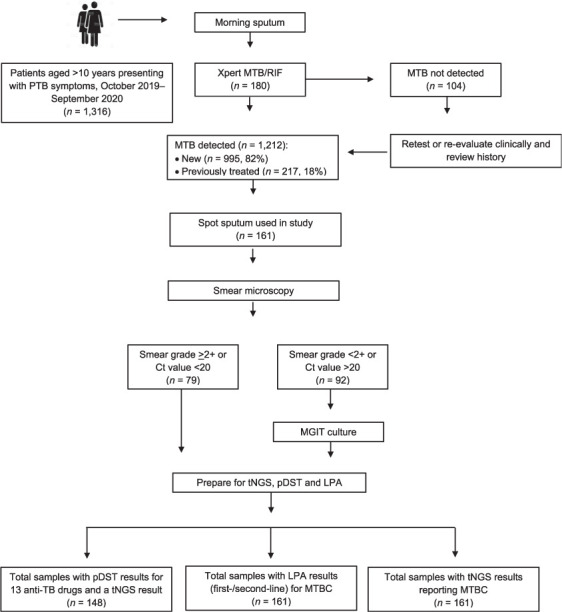
Study design and workflow in patients enrolled for next generation sequencing of drug-resistant TB, Mumbai, India, 2019–2020. MTB = Mycobacterium tuberculosis; PTB = pulmonary TB; Ct = cycle threshold; MGIT = Mycobacteria Growth Indicator Tube; tNGS = targeted next-generation sequencing; pDST = phenotypic drug susceptibility testing; LPA = line-probe assay; MTBC = M. tuberculosis complex.

Bedaquiline susceptibility testing was not performed as it was not yet used in routine care in India. To ensure sequence quality, Xpert results with cycle threshold (Ct) values <20 or direct smear grades >2+ were prepared for tNGS using direct sputum sediments. Primary cultures were prepared for samples with lower bacterial loads. The tNGS reports were not used for clinical management. All patients were managed according to standard-of-care testing under the national guidelines. Figure 1 outlines the study design and workflow.

### DNA extraction, target amplification and next-generation sequencing

DNA extraction and tNGS were performed as described in the Deeplex-MycTB kit (GenoScreen). Selected gene targets were amplified using ultra-deep sequencing of a single, 24-plexed amplicon mix. Amplicons were purified using Agencourt AMPure XP magnetic beads (Beckman Coulter, Indianapolis, IN, USA) and quantified using Qubit dsDNA BR assay (Life Technologies, Paisley, UK). Paired-end libraries (150 base pair [bp]) were prepared using Nextera XT DNA Sample Preparation kits (Illumina Inc, San Diego, CA, USA) and sequenced on an Illumina MiSeq platform. Resistance information was extrapolated from sequence data using a cloud-based analytical platform provided by GenoScreen,^7^ which was informed by published reference databases of genetic variants associated with drug resistance.^10^–^14^ Samples that did not present a clear resistance pattern required further review by a TB sequencing data specialist during post-sequencing data analysis. Uncharacterised mutations with resistant phenotypes were updated according to recent literature or evidence supporting clinical relevance. No patient-related information was linked with the FASTQ (Welcome Trust Sanger Institute, Hinxton, UK) sequence files uploaded or shared in the analysis process.

### Data management and analysis

Medians and proportions were used to describe the demographic and clinical characteristics. Numbers and proportions were used to summarise the analytic output. Sensitivity and specificity for resistance prediction by drug were calculated (95% confidence interval [CI]) against the gold standard MGIT pDST. Calculations were carried out by including and excluding uncharacterised variants. 95% CIs for sensitivity and specificity were generated using the Wilson score method.^15^ The distribution of lineages and number of pre-XDR-TB and XDR-TB strains were defined according to the new WHO definitions.^16^

### Ethics approval

The study received ethics approval from the Institutional Review Board, Grant Government Medical College, Mumbai, and Sir JJ Groups of Hospitals, Mumbai, India.

## RESULTS

### Demographic and clinical characteristics

A total of 161 samples were included in the study. The median age of patients whose samples were examined was 24 years (interquartile range [IQR] 20–40); 57% of these were female (Table 2). Approximately 70% of cases had no previous history of TB.

**Table 2:** Demographic and clinical characteristics of patients enrolled for next-generation sequencing of drug-resistant TB, Mumbai, India, 2019–2020

Characteristics	*n*	%
Total	161	100
Age group, years, median [IQR]	24 [20–40]	
12–19	47	29
20–29	50	31
30–39	31	19
40–49	11	7
≥50	22	14
Sex		
Male	69	43
Female	92	57
Healthcare institution		
Patients from public sector	108	67
Patients from private practitioners	53	33
Culture at baseline		
Positive	148	92
Negative	13	8
Resistance profile		
Susceptible to all TB drugs	15	9.3
INH-monoresistant TB	1	0.6
FQ-monoresistant TB	2	1.2
Other	1	0.6
RR/MDR-TB	142	88.2
WHO 2020 definitions		
Pre-XDR-TB (FQ or injectable)	74	52.1
XDR-TB (FQ and injectable)	24	16.9
WHO 2021 definitions		
Pre-XDR-TB (FQ)	83	58.5
XDR-TB (FQ + LZD or BDQ*)	13	9.2
Previous TB		
Yes	40	24.8
No	113	70.2
Unknown	8	4.9

* Samples with CFZ (rv0687) cross-resistance, BDQ DST was not done.

IQR = interquartile range; INH = isoniazid; FQ = fluoroquinolone; RR = rifampicin-resistant; MDR-TB = multidrug-resistant TB; XDR-TB = extensively drug-resistant TB; LZD = linezolid; BDQ = bedaquiline; CFZ = clofazimine; DST = drug susceptibility testing.

Of the 161 samples with completed pDST, tNGS and LPA were analysed, of which 88.2% were RR/MDR-TB, 15 (9.3%) were RIF-susceptible and 146 (90.7%) RIF-resistant; as per WHO 2020 definitions,^16^ 58.5% had additional FQ resistance (i.e., pre-XDR-TB) and 9.2% had FQ resistance plus resistance to either LZD or resistance associated with *rv0678* which is known to be cross-resistant with bedaquiline (BDQ) (i.e., XDR-TB).

### Comparing resistance results by methods

**Figure 2:**
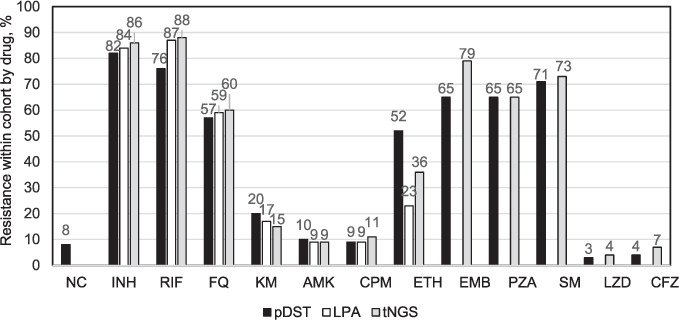
Comparing resistance frequency per method (n = 161) used to evaluate drug resistance (MGIT pDST, Hain LPA and GenoScreen Deeplex MycTB tNGS in patients enrolled for DR-TB testing, 2019–2020. NC = non-culturable; INH = isoniazid; RIF = rifampicin; FQ = fluoroquinolone; KM = kanamycin; AMK = amikacin; CPM = capreomycin; ETH = ethionamide; EMB = ethambutol; PZA = pyrazinamide; SM = streptomycin; LZD = linezolid; CFZ = clofazimine; MGIT = Mycobacteria Growth Indicator Tube; pDST = phenotypic drug susceptibility testing; LPA = line-probe assay; tNGS = targeted next-generation sequencing; DR-TB = drug-resistant TB.

Figure 2 illustrates resistance frequency agreement by method. Overall, tNGS more accurately characterised resistance than pDST, except for resistance to EMB and ETH, which are known to provide unreliable pDST, and not all genetic resistance markers are defined. For drugs with well-established data on resistance-conferring mutations (RIF, FQ, AMK, KM, CPM), resistance agreement was within 5%, except for RIF. Drugs with either borderline phenotypes (e.g., RIF) or limited data on mutations had higher levels of discordance. Although mutations and targets to new and repurposed drugs (LZD, CFZ and BDQ) are not well defined, tNGS detection was more definitive than pDST. For BDQ resistance, only cross-resistance to CFZ (*rv0678*) was defined. A summary of test results for each method (pDST, LPA and tNGS) with listed variants is provided in Supplementary Data 1.

### Performance of targeted next-generation sequencing

Sensitivity and specificity results were compared using pDST as gold standard (Table 3); however, results using tNGS as the reference standard, currently believed to be the direction of the future, are also presented (see Supplementary Data).

**Table 3:** Target-based next-generation sequencing performance by drug in patients enrolled for next-generation sequencing using pDST as the reference standard, Mumbai, India, 2019–2020

**A)** All pDST reporting resistance compared to the specimen’s genotype per each drug					
Drug	Phenotypic-resistant vs. GenoType				

	Resistant	Heteroresistant	Susceptible	Uncharacterised	Total

INH	130		1	1	132
RIF	123	2			125
EMB	89	4		15	108
PZA	90		12	3	105
SM	110		2	2	113
FQ	88	6	4		98
KM	19	1	11	2	33
AMK	14		2		16
CPM	8		5	1	14
ETH	49		16	14	79
LZD	2		2		4
CFZ	2		4		6

All pDST reporting susceptible compared to the specimen’s genotype per each drug					

Drug	Phenotypic-susceptible vs. GenoType				

	Resistant	Heteroresistant	Susceptible	Incomplete coverage	Total

INH	1		15		16
RIF	11	3	14		28
EMB	19		25		44
PZA	2		40		42
SM	3		31		34
FQ	1		55		56
KM	3		109	4	116
AMK			129	3	132
CPM	8		119	7	134
ETH	3		64		64
LZD	1		142	1	142
CFZ	6	1	134		134

**B)** Sensitivity and specificity is reported in this table under two considerations 1) including uncharacterised variants and 2) excluding the uncharacterised variants in the calculations.				

Drug	Including uncharacterised variants		Excluding uncharacterised variants	
	
	Sensitivity % (95% CI)	Specificity % (95% CI)	Sensitivity % (95% CI)	Specificity % (95% CI)

INH	99.2 (95.8–99.9)	93.2 (71.7–98.9)	99.2 (95.8–99.9)	93.2 (71.7–98.9)
RIF*	100.0 97.0–100)	56.6 (37.0–73.3)	100.0 97.0–100)	56.6 (37.0–73.3)
EMB^*†^	100.0 (96.4–100)	56.8 (42.4–70.3)	100.0 (95.9–100)	56.8 (42.4–70.3)
PZA^†^	88.6 (81.1–93.3)	95.2 (84.2–98.7)	88.2 (80.5–93.1)	95.2 (84.2–98.7)
SM	98.2 (93.8–99.5)	91.2 (77.0–97.0)	98.2 (93.7–99.5)	91.2 (77.0–97.0)
FQ	95.7 (89.4–98.3)	98.2 (90.6–99.7)	95.7 (89.4–98.3)	98.2 (90.6–99.7)
KM	65.6 (48.3–79.6)	97.3 (92.4–99.1)	63.3 (45.5–78.1)	97.3 (92.4–99.1)
AMK	87.5 (64.0–96.5)	100.0 (97.1–100)	87.5 (64.0–96.5)	100.0 (97.1–100)
CPM	64.3 38.8–83.7)	93.7 (88.1–96.8)	61.5 (35.5–82.3)	93.7 (88.1–96.8)
ETH^†^	79.7 (69.7–87,1)	95.5 (87.6–98.5)	75.4 (63.7–84.2)	95.5 (87.6–98.5)
LZD^‡^	50.0 (15.0. 85.0)	99.3 (96.2–99.9)	50.0 (15.0. 85.0)	99.3 (96.2–99.9)
CFZ^‡^	33.3 (9.7–70)	96.8 (90.1–98.0)	33.3 (9.7–70)	96.8 (90.1–98.0)

* Low specificity due to low level resistance (borderline).

† High level of uncharacterised variants identified.

‡ Limited information on resistance variants or other resistance mechanisms.

pDST = phenotypic drug susceptibility testing; INH = isoniazid; RIF = rifampicin; EMB = ethambutol; PZA = pyrazinamide; SM = streptomycin; FQ = fluoroquinolone; KM = kanamycin; AMK = amikacin; CPM = capreomycin; ETH = ethionamide; LZD = linezolid; CFZ = clofazimine; CI = confidence interval.

Sensitivity and specificity data were calculated both with and without uncharacterised variants. However, the degree of difference was negligible. Low specificity in RIF is attributed to nine samples harbouring low-level (borderline) mutations. Similarly, there were 20 discrepancies for EMB, a bacteriostatic agent known to give unreliable pDST results, which may have affected overall specificity. Low sensitivities for LZD and CFZ are due to low representation, and the fact that there remains limited information on resistance-conferring mutations for these drugs. In addition, some gene targets are not amplified in the current version of the MycTB kit and therefore go undetected.

Sensitivities (exclusive of uncharacterised variants) were as follows: INH (99%), RIF (100%), EMB (100%), PZA (88%), SM (98%), FQ (96%), KM (63%), AMK (88%), CPM (62%), ETH (76%), LZD (50%) and CFZ (33%); these are in line with WHO catalogue, WHO Global Laboratory Initiatives LPA guide and the initial WHO NGS sequencing guidance document released in 2018.^17^–^19^ Heteroresistance was identified for RIF (1.6%), EMB (3.8%), FQ (6.5%) and KM (3.2%). Significant numbers of uncharacterised variants were noted for EMB (9.9%) and ETH (9.6%) with resistant phenotypes.

In some samples, targets exhibited “I” or insufficient coverage of a gene target related to a specific drug. Most of these were linked to the injectables and LZD targets. The coverage of targets is often related to the quality of the specimen and/or the extracted DNA. Finally, as 13 samples did not grow within the 44-day endpoint for primary culture, no pDST was completed. However, tNGS from direct sputum testing gave resistance profiles for these samples, with 8/13 having resistance to all first-line drugs, SM, FQ and additional resistance to other companion drugs.

### Lineages observed and their relationships to resistance

Table 4 describes resistance across lineages. About 75% of strains identified within Lineage 2 (Bejing, 55%) or Lineage 3 (Delhi/Central Asian strain [CAS], 20%) contributed to 68% RR/MDR-TB, 88% pre-XDR-TB and 65% XDR-TB cases. Of 161 cases, 2 (1%) had mixed infections (Supplementary Data 1). Around 10% (16/161) of the strains harboured heteroresistance. Bejing-type lineages carried hetero-resistance to EMB (2/4), FQ (4/6) and KM (1/1) drugs, while Delhi/CAS type was noted for RIF (3/5).

**Table 4:** Resistance across lineages in patients enrolled for next-generation sequencing of drug-resistant TB, Mumbai, India, 2019–2020

Lineages (*n =* 161)	*n*	%	WHO 2021 definition		
			MDR-TB *n*	Pre-XDR-TB *n*	XDR-TB *n*
Lineage 1: EAI (East African Indian)	17	10.6	11	2	1
Lineage 2: Beijing	88	54.7	84	62	9
Lineage 3: Delhi/CAS (Central Asian strain)	32	19.9	25	11	2
Lineage 4.3: Euro-American LAM (Latin American Mediterranean)	8	5.0	8	2	0
Other than H37Rv	14	8.7	13	5	1
Undefined*	2	1.2	1	1	0

* Lineage 1 (86.9%): 7 (*M. tuberculosis*), 5, 6 (*M. africanum*), animal lineages or *M. canettii* (14.1%).

MDR-TB = multidrug-resistant TB; XDR-TB = extensively drug-resistant TB.

## DISCUSSION

Our study results indicate that tNGS can be used to accurately characterise resistance at a higher frequency than pDST for drugs with well-established data on resistance-conferring mutations (INH, RIF, FQ and injectables). The sensitivity of tNGS in detecting drug resistance was consistent with that reported in the literature,^20^ except for LZD and CFZ, which had low representation in the cohort. However, despite the current evidence-based limitation, our study highlights the potential for tNGS as a more rapid method for resistotyping across all drugs.

For new and repurposed drugs, lack of mutational evidence limits tNGS capability as an independent diagnostic. Thus, countries remain reliant on pDST for these drugs. Unfortunately, pDST testing has not been deployed successfully for all relevant drugs, leaving serious gaps in resistance detection. Although knowledge on resistance-conferring mutations and targets to newer and repurposed drugs are still evolving;^21^,^22^ tNGS predicted resistance more frequently than pDST in this study. High rates of pre-XDR-TB (58.5%) and increasing rates of XDR-TB (9.2%) were observed.

In 2019, the WHO reprioritised anti-TB drugs into new groups and updated the treatment recommendations for the management of DR-TB based on new evidence on effectiveness and safety of new and repurposed drugs.^23^ The definitions of pre-XDR- and XDR-TB were revised to reflect these updates.^16^ To build effective, individualised treatment regimens for DR-TB, technologies that can rapidly and accurately identify resistance for all currently recommended drugs are needed.

Heteroresistance, that is, the coexistence of susceptible and resistant bacilli in the same patient, is problematic for case management if not defined.^24^ Identifying heteroresistance suggests a preliminary stage toward advancing to a full-resistance case, particularly if mismanaged. Individuals with mixed infections, that is, those simultaneously infected with multiple distinct strains of *Mycobacterium tuberculosis*, can also be problematic when one strain dominates the culture, leading to a misrepresented and inaccurate pDST. Both mixed infection and heteroresistance pose serious treatment challenges.^25^ Currently recommended diagnostic methods do not fully address these two in vivo phenomena. Rapid molecular tests have limitations when the number of resistant genomes in a sample falls below the detection threshold of the test. Discrepancies observed between conventional pDST vs. rapid molecular tests can lead to wrongly interpreted results and mismanaged care. Within the Mumbai cohort, 10% of cases harboured heteroresistance at levels of 6% or less. In a patient with a heteroresistance cell population that is lower than the limit of detection on a rapid molecular test, resistance will go undetected, and the patient will essentially fail treatment while spreading resistant disease and acquiring additional resistance to other drugs in the regimen.

Low-level resistance (borderline) mutations were observed, particularly for RIF. Borderline resistance is known to foster discrepancies and thus, inaccuracies that drive misdiagnosis. Mutations that confer borderline resistance are consequently difficult to confirm using pDST (e.g., EMB and RIF). Molecular detection is therefore considered the gold standard. Furthermore, discrepancies between pDST and LPA are often resolved with sequencing.^19^ As currently recommended, rapid molecular assays do not cover all known resistance-conferring mutations. For example, I491F is found outside of the 81 bp RIF resistance determining region of *rpo*B and currently remains undetected. These strains have therefore been genetically selected to remain in circulation and untreated. Using tNGS after an Xpert MTB-positive result is obtained would eliminate misdiagnosis and extensive community transmission. Using an assay with limited gene targets allows patients to go untreated or even worse, develop advanced resistance due to inappropriate therapy. This is where tNGS provides a significant advantage.

Current discussions on epistatic events aim to explain discordant results and the reversion of resistance, which occurs when a resistant mutation is present, but a downstream mutation causes a loss of function (LoF) such as an indel. Essentially, the LoF mutation negates the effect of the resistance mutation, which triggers a false-resistant call. In case of CFZ for example, this may be reflective of mmpR (*rv0678*) mutations that are not valid if they coincide with LoF mutations (indels) of *mmpS5* and *mmpL*.^26^

For the baseline culture performed for all samples in preparation for pDST, 13 samples were culture-negative. Of these, eight provided tNGS results (from direct sputum) that were resistant to all first-line and various second-line drugs. The cause of the poor cultivation of these samples is generally severe drug-damage or altered cell metabolism, making the cells viable but non-culturable (VBNC) in liquid medium.^27^ This poses serious implications for patient care and results in possible under diagnosis, as pDST is reported as “no growth”, leading to either no treatment or empirical treatment of patients. This phenomenon can lead to an underestimation of the total burden of DR-TB and pose a transmission risk in the community.

A key factor in the production of a quality sequence with good coverage of all targets depends on the quality of either the sputum specimen or the genomic DNA post extraction. Having enough bacilli (MTB genomes) for target amplification is critical for any molecular assay. For example, in our study several gene targets reported “I” or “insufficient coverage” across a gene target (i.e., *rrs*, *rrl* and rplC). Low coverage leads to unreportable results, which makes decision-making difficult for the use of some drugs (in our case, AMK or LZD). Therefore, the quality of the sample, as with most diagnostics, is the key to accurate results.

Viola Dreyer et al. reported high rates of pre-XDR-and XDR-TB strains in association with Lineage 2 (Beijing) in Mumbai.^28^ The study also reported Lineage 2 as the dominant strain carrying the highest likelihood for DR-TB. Lineage 2 has also been associated with higher virulence and faster progression to disease.^28^ Another study reported children exposed to Beijing strains were more likely to be infected and develop disease.^29^ Thus, understanding circulating genotypes can be key to epidemic control. tNGS can be used to monitor trends in strain prevalence and provide a better understanding of the disease within the community, allowing TB programmes to alter guidance and practices as needed when more infectious, virulent or resistant strains begin to dominate circulation. This is most relevant for countries with high transmission rates. In Mumbai, 70% of cases in our cohort acquired resistance through community transmission.

Increases in BDQ resistance and resistance to repurposed drugs (LZD/CFZ) are being reported worldwide.^21^,^22^ To support the goal of rapid diagnosis and effective treatment in high DR-TB burden settings, tNGS placement in existing diagnostic algorithms should be considered. This should be determined by national epidemiology based on resistance rates (not limited to RR-TB alone), as well as rates of transmission within the population served.

The NTEP rolled out BDQ under a conditional access programme (CAP) in 2016 at six sites,^30^ which was later expanded to all sites. In the Indian context, the NTEP algorithm presently recommends LPA (first- and second-line) testing for rapid detection of INH, RIF, FQ and AMK resistance, followed by pDST for LZD and high-dose MFX.^31^ Neither CFZ nor BDQ pDST are yet to be implemented as routine care. Routine surveillance and testing are necessary for efficient identification of any evolving BDQ resistance before the strain enters circulation in the population. Once in circulation, these could have serious and devastating implications, such as limiting successful treatment options for care. As Mumbai is a city of migrants and densely populated, BDQ transmission in such a place poses significant risk. About 70% of cases in our study showed acquired resistance through community transmission; upfront use of tNGS for all MTB-positive cases detected using Xpert should therefore be considered.

The study had some limitations. The non-availability of pDST for BDQ and capturing of only one mutation (*rv0678*), i.e., cross resistance between CFZ and BDQ led to limitations in the accuracy of resistance prediction for BDQ. As external quality assessment of DST was available only for drugs included in the current diagnostic algorithm under the national guideline, pDST for additional drugs may be unreliable. The high proportion of DR-TB is not reflective of the Mumbai East Ward cohort, as a protocol change to select MTB RIF-positive specimens over MTB RIF-susceptible specimens was made during enrolment to collect more specimens with second-line resistance. This was to better understand more about what second-line mutations were currently in circulation

## CONCLUSION

In general, study data support the use of direct sputum tNGS for resistance-prediction profiling for most commonly used drugs. Rapid resistotyping could provide enough information to design regimens for early and more effective treatment, leading to better treatment outcomes for patients with DR-TB and prevention of the transmission of highly evolved TB strains.
